# Physicians and Life Insurance Companies

**Published:** 1895-09

**Authors:** 


### Editorial.

### PHYSICIANS AND LIFE INSURANCE COMPANIES.

The business of life insurance has within the last two decades grown to enormous proportions, and the relation of the physician to the companies as medical examiner, is a most important one. All life insurance is based upon reliable data, this data having been obtained by careful study and observation of the life history of large numbers of *healthy individuals*. Necessarily then, such data must prove unreliable, and an unsafe guide if any considerable number of policy holders are unsound at the time the policy is issued. To provide against such a possibility, the companies have in every town and city medical examiners whose duty it is, acting in the interest of the company, to make a careful physical examination of all applicants, to obtain from them their own and their family histories and to make a chemical examination of their urine. Based upon such examination, the local examiner makes a report to the home office. It is easy, then, to see that back of all safe insurance, and as an absolute prerequisite to it, there must be both capable and honest local medical examiners; men of sufficient ability and judgment to make nice distinctions and arrive at just conclusions. The companies, recognizing these facts, have endeavored, in towns and cities, to obtain as examiners physicians of recognized ability and unquestioned integrity, and knowing that the services of such men can only be obtained for a fair and assured compensation, have without exception paid a fee of five dollars for each examination made —we refer only to what are known as "old line" companies. This amount, though less than would be charged in the larger cities for similar work in private practice, is more than would be customary in towns and villages, and possesses the advantage of certain payment; hence such a fee has generally become recognized, by both the companies and the profession, as a fair compensation for an honestly conducted examination.

Now one of the oldest and largest companies, viz.: The New York Life, comes to the front and declines to pay their examiners more than three dollars an examination. Will the profession accept it? We think not. We recognize the right of the company to get its examinations made as cheaply as possible, but we recognize equally the right of the physician to demand fair compensation for his services. It seems pertinent to ask whether or not the company has reduced the salaries of its president and lesser officials, whether its agents get less commissions, whether or not its premium rates have been lowered or its dividends increased. If not, who is to profit by the reduced examiner's fee. As the experience of all companies shows, there will, with the utmost care, always slip in some bad risks. Will this number be lessened by securing as examiners, men who are either willing to work for less than a fair compensation, or who from necessity are forced to take what they feel to be inadequate remuneration, and will such examiners, though honest men, be as careful and as painstaking as when paid fairly for their work? Suppose an examiner made twenty examinations a month, for which he would receive, at five dollars for each one, one hundred dollars—for this, he would have expended twenty hours of time, prepared carefully twenty written reports, made twenty examinations of the chest, twenty chemical examinations of urine, and been annoyed by officious agents who wished the physician to drop everything and make an examination at once, etc. In the large companies, the average amount of a policy is, in round numbers, three thousand dollars; twenty policies would, then, represent a liability upon the part of the company of sixty thousand dollars; for passing judgment upon which, the examiner would have gotten one-sixth of one per cent, the soliciting agent, at an expenditure of more time but less skill and no responsibility, would have received, for his work, from one to one and one-half per cent of the amount insured. Remember, the expense of an examination occurs but once in the life of a policy. Now, what would be the charges of an attorney for the examination of the title of property involving a similar amount; or to come nearer home, what would be paid an actuary for similar work.

Lastly, upon twenty examinations, the company would, at the proposed fee of three dollars, save forty dollars. One carelessly or incompetently made examination might cost the company nearly all of three thousand dollars.

We sincerely trust that the protection afforded widows and orphans by safe insurance, may not be jeopardized by a short-sighted policy which aims to save at the spigot while disregarding the bung. L. H. J.

---

Dr. D. H. Howell, formerly business manager of the SOUTHERN MEDICAL RECORD, has accepted a position with Reed & Carnrick. We wish Dr. Howell much success, and a rapid restoration to health.

---

The Cotton States and International Exposition will be formally opened by President Cleveland, on September 18. In size, picturesqueness of location, and general excellence of the exhibits, it bids fair to be a lasting monument to the good taste, thrift and enterprise of Atlanta. Rates on all the railroads coming into Atlanta have been very much reduced, and there will be ample accommodations for visitors in the city. Dr. George Brown has very kindly offered to make his office the headquarters of visiting professional brethren. Any letters of inquiry, containing postage stamps for reply, will be promptly answered, and rooms engaged without commission. The same courtesy will be extended by us, if letters are addressed to this office.

---

### TROUBLES IN THE COMMUNITY.

The coal dealer died of colitis,
  The twine maker had the chord-ee.
The farmer's attack of oat-itis
  And rye-neck was painful to see;
The wheelman went blind with cyclitis.
  The bridge builder suffered from piles.
The servant girl had Sal-pingitis,
  And the cook was all covered with b'iles.

